# Optimization and Immobilization of Purified *Labeo rohita* Visceral Protease by Entrapment Method

**DOI:** 10.1155/2013/874050

**Published:** 2013-02-27

**Authors:** S. Geethanjali, Anitha Subash

**Affiliations:** Department of Biochemistry, Biotechnology and Bioinformatics, Avinashilingam Institute of Home Science and Higher Education for Women, Coimbatore, Tamil Nadu 641 043, India

## Abstract

The purified fish visceral protease enzyme was immobilized by using various concentrations of sodium alginate and calcium chloride to optimize the best concentration for the formation of the beads. Then it was characterized by assaying the optimal pH, temperature, storage stability and reusability. The results on immobilization with sodium alginate and calcium chloride showed that a combination of 2% sodium alginate and 0.3 M calcium chloride weas found to be the optimum concentration for the formation of spherical and stable beads, this gave a maximal entrapped activity of 48.31%, and there was no change in the optimum pH 8.0 and temperature 40°C of protease before and after entrapment. The results on stability and reusability indicated that it was stable at 4°C retaining 100% residual activity after 5 days of storage and 67% loss of activity after ten days of storage and it retained 100% residual activity on the first reuse, 75% residual activity on the second reuse, 25% residual activity on the third use and complete loss in the activity on the fourth reuse.

## 1. Introduction

*Labeo rohita*, commonly called Rohu, is a member of the family Cyprinidae within the order Cypriniformes [1]. It is a prime cultured and important staple fresh water fish generally found in rivers, ponds and reservoirs [2]. In recent years, recovery and characterization of enzymes such as alkaline phosphatase, hyaluronidase, acetylglucosaminidase, chitinase, and protease from fish have been carried out, and these have led to the emergence of some interesting new applications of these enzymes in food processing [3, 4].

Protease is an enzyme which hydrolyses proteins, that is, catabolizes proteins by hydrolysis of the peptide bonds that link amino acids together in the polypeptide chain forming the proteins. Proteases also known as peptidyl-peptide hydrolase constitute 60–65% of the global enzyme market [5, 6]. Proteases are mainly derived from animal, plant, and microbial sources. They play an essential role in the growth and survival of all living organisms. For marine animals, proteases are mainly produced by the digestive glands. The most important digestive enzymes are pepsin, secreted from gastric mucosa; trypsin and chymotrypsin secreted from the pancreas, pyloric caeca, and intestine. Like the proteases from plants, animals and microorganisms, digestive proteases from marine animals, are polyfunctional enzymes catalyzing the hydrolytic degradation of proteins [7].

The protease enzyme has diverse applications in a wide variety of industries such as detergent, food, pharmaceutical and leather industries, peptide synthesis and for the recovery of silver from used X-ray films [8]. Use of enzymes as catalysts for large-scale industrial processes is limited by their high cost of production and stabilization on storage. During use, their stability decreases due to changes in pH, temperature, conformational changes as a result of friction, osmotic pressure imposed by the environs of their use, and a cumulative effect of all these factors as a function of duration of their use. Secondly, since they are soluble, their recovery from a mixture of substrate and product for reuse is not economically practical rendering the costly enzymatic process even more costly. However, the advent of immobilized enzyme technology has led to increasing efforts to replace conventional enzymatic processes with immobilized preparations [9].

The general methods employed for immobilization are entrapment, microencapsulation, copolymerization, cross linking, physical adsorption, chemical attachment, and covalent binding [10]. Among different immobilization techniques entrapment in calcium alginate gel offers many advantages due to its simplicity and nontoxic character [11]. 

Usually, microbial alkaline protease dominates the world enzyme market. Hence a trial has been carried out to use fish visceral protease as an alternative source for the microbial protease. The present study exploited the simple technique of entrapment using calcium alginate for the immobilization of purified protease from the viscera of *Labeo rohita* which is abundantly consumed by populations of Salem, Tamil Nadu, India.

## 2. Materials and Methods

### 2.1. Analytical Reagents

Bovine serum albumin, casein, trichloroacetic acid, Folin-Ciocalteu's reagent, sodium carbonate, and Tris(hydroxylmethyl) aminomethane were purchased from Himedia, Mumbai, India. Sodium alginate and calcium chloride were purchased from Loba Chemie, Mumbai, India.

### 2.2. Preparation of Enzyme Solution

*Labeo rohita* were purchased from the local market in Mettur Dam, Tamil Nadu, India. The fish were kept in ice and transported to the research laboratory within 1 hour. After washing the fish with distilled water, visceral organs were separated and then stored in sealed plastic bags at −20°C until it is used for enzyme extraction. Viscera from *Labeo rohita* were thawed for about 2 hours at room temperature, and 80 g of fish intestine and pyloric caeca were weighed and homogenized with 250 mL of 10 mM tris-HCl buffer (pH 8.0) [12]. The homogenate was centrifuged at 8500 ×g for 30 min at 4°C. The pellet was discarded and the supernatant was collected and used as crude protease extract. Further, it was purified by Sephadex G-100 and DEAE cellulose column chromatography.

### 2.3. Assay of Protease Activity

Protease activity was assayed by the Anson method [13] with some modifications. The enzyme solution (1 mL) was mixed with 5.0 mL of substrate (0.65% casein in 25 mM tris-HCl buffer, pH 8.0) at room temperature for 30 min. After incubation, TCA (110 mM) was added to attenuate the reaction. This mixture was allowed to incubate for 30 min at room temperature and filtered to remove the precipitate. The absorbance was measured at 660 nm. A standard curve was generated using solutions of 0.2 mg/mL tyrosine. One unit will hydrolyze casein to produce colour equivalent to 1.0 *μ*mole (181 *μ*g) of tyrosine per minute at pH 8.0 at 37°C.

### 2.4. Enzyme Immobilization

The enzyme immobilization was performed according to Chellapandi [14]. The purified fish visceral protease was immobilized with sodium alginate and calcium chloride. A 2% sodium alginate solution was prepared by dissolving sodium alginate in 100 mL hot water. The contents were stirred vigorously for 10 min to obtain thick uniform slurry without any undissolved lumps. 

The purified fish visceral protease enzyme solution was mixed with sodium alginate solution (2%) in 1 : 1 ratio. The protease-alginate mixture was added dropwise into calcium chloride (0.3 M) solution from 0.5 cm height and kept for curing at 4°C for 1 hr. The cured beads were recovered by filtration and thoroughly washed with distilled water and finally with 25 mM tris-HCl buffer of pH 8.0. 

### 2.5. Determination of Immobilized Protease Activity

 The activity of immobilized enzyme was assayed by incubating 5 mL of 0.65% (w/v) casein solution (prepared in 25 mM tris-HCl buffer of pH 8.0) with 0.5 g of immobilized calcium alginate beads at 25°C for 30 min. After the enzymatic reactions had proceeded, 5 mL of 110 mM TCA was added to terminate the reaction, and then it was assayed for protease activity using tyrosine according to the Anson method [13]. Immobilization efficiency was evaluated by using the following the formulae [15]:

 initial activity of the free enzyme = “*a*”,
 volume of enzyme solution = “*b*”,
 weight of beads formed after immobilization of enzyme solution = “*c*”,
 enzyme solution entrapped in 0.5 g beads = *b/c* × 0.5 =“*d*”,
 based on the initial activity of the free enzyme,
 activity of the enzyme entrapped must be = *a *× *d*/1 mL =“*e*”,
 activity of immobilized enzyme obtained in 0.5 g beads = “*f*”,
 therefore total enzyme activity after entrapment = “*X*”%.

### 2.6. Optimization of Immobilized Condition

 Immobilization parameters should be studied and optimized to preserve the native activity of biomolecules and to achieve high immobilization efficiency [16]. For this purpose varying concentrations of sodium alginate (1–5% w/v) and calcium chloride (0.1–0.5 M) were used during immobilization of fish visceral protease to achieve 100% immobilization yield.

### 2.7. Characterization of Immobilized Protease

#### 2.7.1. Effect of pH and Temperature

The optimum pH for the immobilized and free protease was determined using the substrate casein prepared in buffers of varying pH ranging from 4.0 to 12.0 (50 mM sodium acetate buffer, pH 4.0–6.0; 50 mM phosphate buffer, 7.0; 50 mM Tris-HCl buffer, 8.0-9.0; 50 mM Glycine-NaOH buffer, 10.0–12.0).

 To determine the optimum temperature of free and immobilized protease enzyme, activities were measured in the temperature range of 20–80°C. The relative activity was calculated in percentage with reference to the activity of the optimum pH or temperature (100%).

### 2.8. Storage Stability of Immobilized Enzyme

 The storage stability was investigated for both soluble and immobilized enzyme preparations at 4°C and 25°C. These temperatures were selected based on two criteria. Generally, for short term storage like one day or a few weeks many proteins are stored in simple buffers at 4°C. At 25°C the isolated soluble enzyme was found to be very active, and there was no necessity of any special preservation method to store the enzyme. Hence, for these reasons the above mentioned temperatures were selected. The stored immobilized enzyme was assayed every day for a period of 10 days to determine its activity. The residual activities were calculated based on the initial activity.

### 2.9. Reusability of Immobilized Protease

The reusability of the immobilized protease was determined by standard assay conditions, and the activity was checked every day till there was no activity at all. The residual activity was calculated by taking the enzyme activity of the first cycles as 100%. 

### 2.10. Statistical Analysis

The data obtained from the studies in optimization on immobilization conditions were analyzed by one-way ANOVA and DMRT to find out the statistically significant differences in the parameters studied among the different treatment groups.

## 3. Results and Discussion

### 3.1. Optimization of Immobilization Parameters

For the preparation of beads with proper permeability and rigidity, parameters such as sodium alginate concentration and molarity of the calcium chloride need to be optimized. The following parameters depict the immobilization efficiency of the purified protease from *L. rohita* viscera.

#### 3.1.1. Effect of Sodium Alginate Concentration

Among all immobilization matrices studied, sodium alginate attracted scientific attention due to its eco-friendly nature, cost-effectiveness, mild conditions required for immobilization, simplicity, and nontoxic nature [17] The degree of cross-linking of the gelling agent affects the pore size of the beads, and therefore various concentrations of sodium alginate were used to achieve the highest immobilization efficiency [18].

The pore size of the beads should be such that the substrate and product easily diffuse in and out of the alginate gel matrix retaining the enzyme in the micro environment of the beads. The lower the concentration of sodium alginate solution, the greater will be the pore size of the beads resulting in increased leakage of the enzyme from the beads. Similarly, the higher the concentration of sodium alginate, the smaller the pore size of the beads leading to lower immobilization efficiency [19]. 

The effect of sodium alginate concentration ranging from 1 to 5% and 0.2 M calcium chloride on immobilization of purified fish visceral protease of *L. rohita* is shown in Table 1.

The data from the table confirm that the significantly (*P* < 0.05) highest immobilization (45%) of the isolated enzyme was obtained with beads prepared from 2% w/v of sodium alginate. After this, there was a gradual decrease in the immobilization efficiency with 5% sodium alginate recording a lower percentage immobilization. The lowest percent immobilization was observed with 1% sodium alginate. This may be due to maximum leakage of enzymes from the immobilized beads due to the larger pore sizes of the beads and the less tight cross links with the calcium chloride which results in a lower immobilization percentage of 10.

Immobilization with 2% sodium alginate as observed from the present study is also supported by the work of Farag and Hassan [20] who stated that sodium alginate ranging from 2 to 3% was suitable for immobilization of keratinase, lipase, and protease. In the case of increased sodium alginate concentrations such as 3%, 4%, and 5%, the entrapped enzyme activity was found to be lower since the pore sizes of the beads decreased. This is in accordance with the results of Dey et al. [19] who observed that increased sodium alginate concentration interferes with the entry of substrates into the beads which ultimately leads to lower immobilization efficiency. 

Adinarayana et al. [21] also found that an increased alginate concentration leads to reduced porosity of the beads, thereby limiting the nutrient supply and oxygen diffusion in whole cell immobilization of *B. subtilis* PE-11.

#### 3.1.2. Effect of Calcium Chloride Concentration

Calcium chloride is used as a cross linking agent, and its concentration affects the activity and density of immobilized cells [22]. The mechanical strength of alginate beads is highly dependent on the calcium chloride concentration of the gelation solution, and the use of concentrated calcium chloride solution has a higher effect on the efficiency of immobilized systems [23]. Thus the concentration of calcium chloride is important for the stability and pore size of the bead [24].

Alginate beads were prepared with varying concentrations of calcium chloride (0.1, 0.2, 0.3, 0.4, and 0.5 M) mixed with 2% sodium alginate since 2% showed the highest immobilization efficiency. 

The effect of calcium chloride concentration on the rigidity of the beads is shown in Table 2. From the table, it is obvious that calcium chloride at a concentration of 0.3 M registered the highest entrapped enzyme activity with the percentage immobilization value being 48.3. This was significant (*P* < 0.05) when compared to the beads prepared using 0.1 M and 0.2 M calcium chloride, revealing an entrapped enzyme activity of only 29% and 36%, respectively. This concentration also forms beads which are irregular in shape. In the case of beads prepared with 0.4 M and 0.5 M calcium chloride exhibiting percent immobilization of 31% and 24%, respectively were disintegrated at the end of the immobilization assay.

As for sodium alginate, lower concentrations of calcium chloride may possibly have resulted in increased leakage of enzymes. The finding that a calcium chloride concentration of 0.3 M was found to be the optimum for the immobilization of purified protease can be correlated with the reports of Ahmed et al. [25], who stated that immobilization of cells using 0.3 M calcium chloride was found to be the best for the enzyme invertase from *Bacillus macerans*.

### 3.2. Effect of pH on Activity of Immobilized Protease

**Figure 1:**
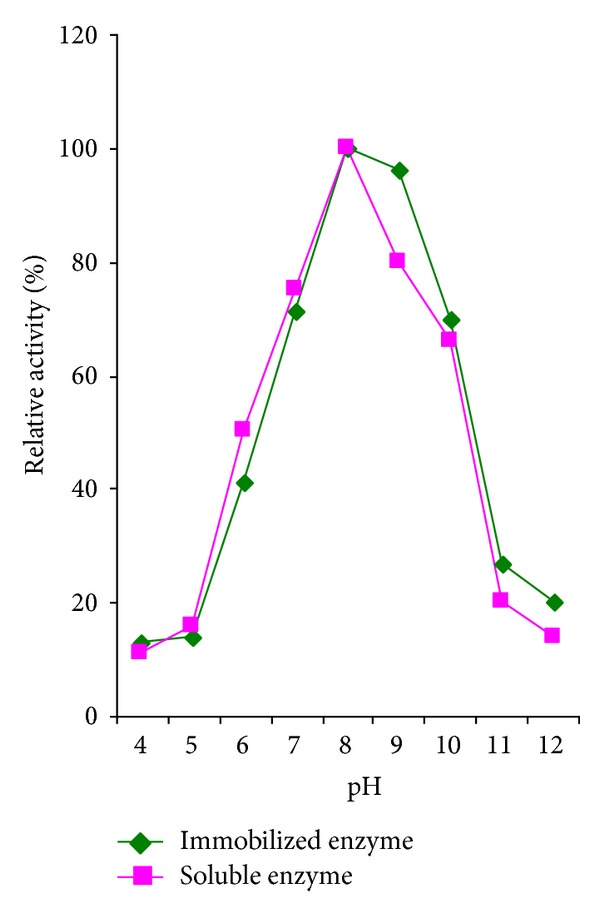
Effect of pH on immobilized fish visceral protease.

The effect of pH on the relative activity of the immobilized and free protease of *L. rohita* viscera is compared in Figure 1. The activity was studied at a pH range of 4.0–12.0 and an optimum temperature of 40°C. From the figure, it is evident, that there was a gradual increase in the enzyme activity from pH 4.0 to pH 8.0 and then a gradual decrease from 8.0 to pH 12.0 which implies that the optimum pH for the activity of the isolated enzyme is 8.0. A similar trend was followed for the free enzyme also. This clearly shows that there is no change in the optimum pH of the enzyme on entrapment. As shown in Figure 1 the pH curves of the immobilized and soluble enzymes were similar except at pH 9.0. This behavior might be the result of activity shift to more alkaline values. 

The alkaline shift for the catalytic activity as an effect of immobilization on anionic matrix (alginate) was in agreement with the general observation of Morana et al. [26] who stated that the negatively charged supports displace pH activity curves towards higher pH values.

Similar findings were observed by Anwar et al. [15] on immobilization of proteases from newly isolated strain of *Bacillus subtilis* KIBGE-HAS where they observed no change in the pH of protease before and after entrapment with calcium alginate. Another study by Arya and Srivastava [27] also reported that there was no change in the optimum pH of cyclodextrin gluconotransferase (CGTase) before and after entrapment in calcium alginate beads.

### 3.3. Effect of Temperature on the Activity of Immobilized Protease

**Figure 2:**
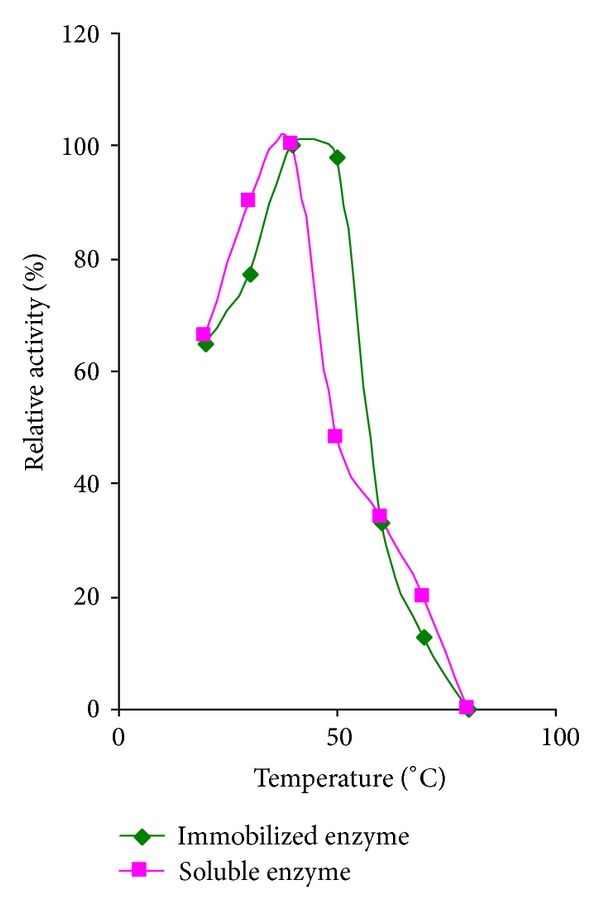
Effect of temperature on activity of immobilized fish visceral protease.

Alginate entrapped enzyme was assayed at different temperatures ranging from 20 to 80°C at an optimum pH of 8.0. The properties of the immobilized and free enzymes are depicted in Figure 2.

From the figure, it is clearly understood that the activity of both soluble and immobilized protease increased from 20°C to 50°C and then decreased up to 70°C. Beyond this temperature, there was absolutely no activity detected. Therefore, it can be deduced that the optimum temperature for the isolated enzyme was 40°C for both the entrapped and soluble forms. However, in the case of the immobilized enzyme even at 50°C there was a high activity of 98% which was not so for the soluble enzyme. The highest relative activities of the immobilized enzyme were observed at a temperature of 40°C (100%), followed by 50°C (98%). As regards the soluble enzyme, the highest relative activity was exhibited at 40°C (100%) followed by 30°C (90%).

Hence a shift to a higher temperature was observed for the activity of the immobilized enzyme with respect to that of the corresponding free form. Hence it was difficult to determine the near optimal temperature for the immobilized and free enzymes. Moreover, it was observed that when the temperature was raised above the optimum value from 40°C to 50°C, the immobilized enzyme still retained 98% of its activity while the free enzyme showed only 48% activity. This shows the significance of immobilization. There may be several reasons responsible for these changes, which includes, three-dimensional structure and activation energy of the immobilized enzyme. Generally, the three-dimensional structure of an enzyme become altered during the immobilization procedure. Similarly, the activation energy of the immobilized enzyme might be lower than the free enzyme.

The above results are supported by Srinivasa Rao et al. [28], who emphasized that immobilization of *α*-chymotrypsin, trypsin, and pepsin on tri(4-formyl phenoxy) cyanurate revealed a shift in optimum temperature from 40 to 50°C for *α*-chymotrypsin and trypsin and 50 to 60°C for papain. Amaral et al. [29], on the other hand, reported that fish trypsin immobilized on ferromagnetic dacron had an optimum temperature of 50°C which was lower than that found for the soluble enzyme at 55°C. 

### 3.4. Storage Stability of Immobilized Protease

Storage stability is a prominent factor for commercialization of an enzyme [30]. Immobilization is a form of storage and it is advisable to immobilize enzymes because free enzymes can lose their activities quickly. In general, if an enzyme is in aqueous solution, it is not stable during storage and the activity gradually reduces [31]. 

**Figure 3:**
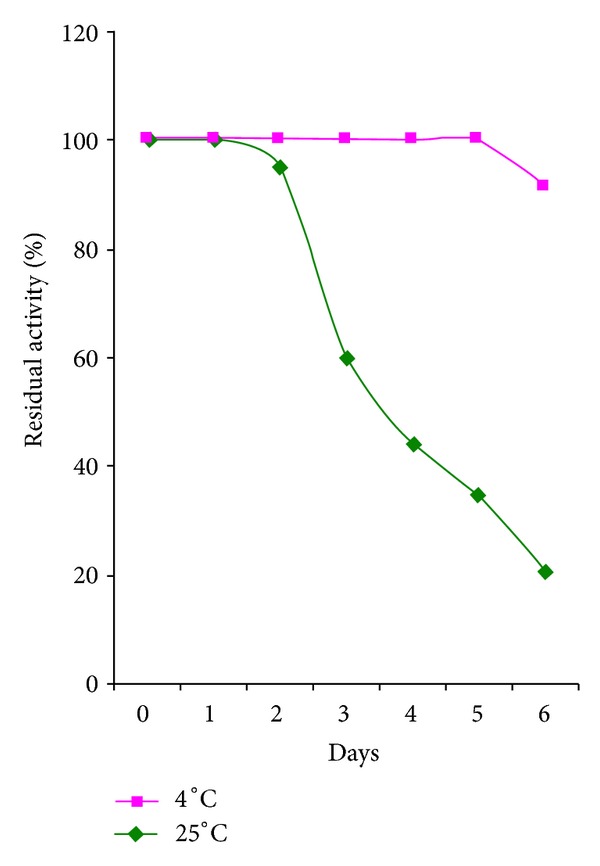
Storage stability of immobilized fish visceral protease at different temperatures.

Figure 3 indicates the storage stability of the immobilized protease from *L. rohita* viscera. The immobilized enzyme was stored at two temperatures (4 and 25°C) and its activity noted everyday for 10 days to determine the storage stability of the entrapped enzyme. These temperatures were selected based on the optimal activity of the isolated protease. It is obvious from the figure that, at 25°C, the immobilized enzyme was stable only for 2 days with a residual activity of 100% on the 0th and 1st day and 95% on the 2nd day. After this, there was a gradual decline in the activity till the 6th day. Beyond this, there was no activity detected at all up to the 10th day. At 4°C, the immobilized enzyme remained stable up to the 5th day with 100% activity and 6th day with 91% activity. After this, there was a progressive fall in the activity up to the 10th day unlike the samples at 25°C the sample studied at 4°C, and showed a higher activity even up to the 10th day. 

The above observations suggest that the enzyme is more stable at 4°C when compared to 25°C, since 4°C is the suitable temperature for storage of most of the enzymes. At 25°C proteases might undergo autolysis because, proteases, being themselves proteins, are known to be cleaved by other protease molecules, sometimes of the same variety, which might be the reason of inactivation of the immobilized enzyme.

The above results reporting decrease in enzyme activity with increase in the number of days of storage are supported by the work of Anwar et al. [15], who stated that calcium alginate beads stored at 4°C showed 35% loss of residual activity after two days and 89% loss of residual activity after ten days. Another study by Qader et al. [32] also reported a 36% loss in the residual activity of immobilized dextransucrase at 30°C after 3 hours and observed 86% loss in residual activity at 40°C within two hours.

### 3.5. Reusability of Immobilized Protease

**Figure 4:**
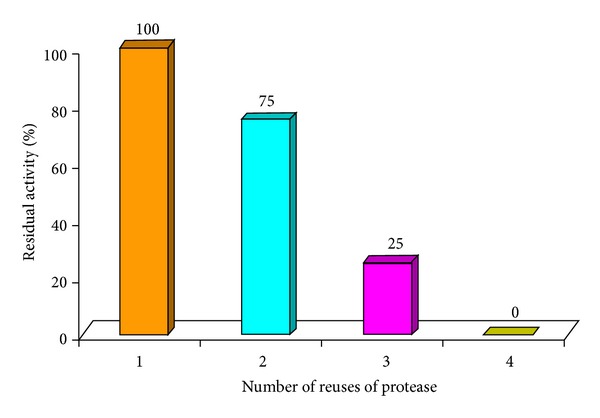
Reusability of immobilized fish visceral protease.

The reuse of the immobilized enzyme is very important from the point of view of reducing the cost of the enzyme. This is an important factor while considering its table suitability for commercial applications [33]. The reusability pattern of immobilized protease is depicted in Figure 4.

From the values in the above mentioned figure, it can be deduced that the entrapped enzyme showed 100% residual activity during the first use, 75% activity during the second reuse, 25% residual activity on the third reuse, and complete loss in the activity on the fourth reuse. During reuse, the immobilized enzyme became dark in colour and showed lower residual activity. This might be the reason for deterioration of the immobilized enzyme. The above results are in accordance with the reports of Pithawala et al. [34] who observed that the free enzyme retained the same activity for about seven days, after which it slowly decreased and the activity was lost completely after a month. The enzyme immobilized on calcium alginate retained the same activity for a fortnight, then decreased and was lost in 30 days. The beads were also darkened (slightly brown) and deteriorated (beads were stored at room temperature in calcium chloride solution). In another study, milk clotting enzyme produced from *Bacillus sphaericus *NRC 24 was immobilized efficiently on silica gel and was found to be active for three reuses and retained 50% of its activity after four cycles. It was also found that there was no leaching of the enzyme after six cycles [35].

However, Anwar et al. [15], who had entrapped protease in calcium alginate beads and reused it up to three times, had reported that the decrease in activity occurred on further reuse which was due to the leakage of enzyme from the beads, during washing at the end of each cycle. Another study stated that alpha amylase entrapped in Ca-alginate beads could be reused for six cycles with about 30% loss in activity [36].

From the above results, it can be concluded that all the concentrations of sodium alginate and calcium chloride showed entrapped activity. A concentration of 2% sodium alginate and 0.3 M calcium chloride was found to be the optimum concentration for formation of spherical and stable beads with maximal entrapped activity of 48.3%. The effect of pH and temperature of the entrapped enzyme showed an optimum of 8.0 and 40°C. The storage stability of the immobilized protease suggests that it was more stable at 4°C when compared to 25°C. The study on reusability experiment concluded a 25% residual activity on third use and complete loss in the activity on the forth reuse.

## Figures and Tables

**Table 1:** Effect of sodium alginate concentration on immobilization of fish visceral protease.

Sodium alginate concentration (%)	% immobilization
1	10 ± 0.35^e^
2	45 ± 1.54^a^
3	33 ± 1.13^b^
4	31 ± 0.95^c^
5	21 ± 0.73^d^

Values are mean ± SD (*n* = 6).

Values in the column not sharing a common superscript letter differ significantly at *P* < 0.05 (DMRT).

**Table 2:** Effect of calcium chloride on immobilization of fish visceral protease.

Calcium chloride concentration (M)	% immobilization
0.1	29.00 ± 1.03^d^
0.2	36.00 ± 1.26^b^
0.3	48.30 ± 1.74^a^
0.4	31.00 ± 1.18^c^
0.5	24..00 ± 0.97^e^

Values are mean ± SE (*n* = 6).

Values in the column not sharing a common superscript letter differ significantly at *P* < 0.05 (DMRT).

## References

[1] Yeasmin T, Reza MS, Shikha FH, Khan MNA, Kamal M (2010). Quality changes in formalin treated rohu fish (*Labeo rohita*, Hamilton) during ice storage condition. *Asian Journal of Agricultural Sciences*.

[2] Dube PN, Hosetti BB (2010). Behaviour surveillance and oxygen consumption in the freshwater fish *Labeo rohita* (Hamilton) exposed to sodium cyanide. *Biotech Animal Husbandry*.

[3] Castillo-Yañez FJ, Pacheco-Aguilar R, Garcia-Carreño FL, Navarrete-Del Toro MDLA (2004). Characterization of acidic proteolytic enzymes from Monterey sardine (*Sardinops sagax caerulea*) viscera. *Food Chemistry*.

[4] Shahidi F, Kamil YVAJ (2001). Enzymes from fish and aquatic invertebrates and their application in the food industry. *Trends in Food Science and Technology*.

[5] Genckal H, Tari C (2006). Alkaline protease production from alkalophilic *Bacillus* sp. isolated from natural habitats. *Enzyme and Microbial Technology*.

[6] Rawlings ND, Morton FR, Kok CY, Kong J, Barrett AJ (2008). MEROPS: The peptidase database. *Nucleic Acids Research*.

[7] Klomklao S (2008). Digestive proteinases from marine organisms and their applications. *Songklanakarin Journal of Science and Technology*.

[8] Nasri R, Younes I, Lassoued I, Ghorbel S, Bellaaj OG, Nasri M (2011). Digestive alkaline proteases from Zosterisessor ophiocephalus, Raja clavata, and Scorpaena scrofa: characteristics and application in chitin extraction. *Journal of Amino Acids*.

[9] Kotwal SM, Shankar V (2009). Immobilized invertase. *Biotechnology Advances*.

[10] Hasirci N, Aksoy S, Tumturk H (2006). Activation of poly(dimer acid-co-alkyl polyamine) particles for covalent immobilization of *α*-amylase. *Reactive and Functional Polymers*.

[11] Gangadharan D, Nampoothiri KM, Sivaramakrishnan S, Pandey A (2009). Immobilized bacterial *α*-amylase for effective hydrolysis of raw and soluble starch. *Food Research International*.

[12] Barkia A, Bougatef A, Nasri R, Fetoui E, Balti R, Nasri M (2010). Trypsin from the viscera of Bogue (Boops boops): isolation and characterisation. *Fish Physiology and Biochemistry*.

[13] Anson ML (1938). The estimation of pepsin, trypsin, papain and cathepsin with hemoglobin. *The Journal of General Physiology*.

[14] Chellapandi P (2007). *Laboratory Manual in Industrial Biotechnology*.

[15] Anwar A, Qader SAU, Raiz A, Iqbal S, Azhar A (2009). Calcium alginate: a support material for immobilization of proteases from newly isolated strain of *Bacillus subtilis* KIBGE-HAS. *World Applied Sciences*.

[16] Lu SY, Qian JQ, Wu ZG (2009). Application of statistical method to evaluate immobilization variables of trypsin entrapped with sol-gel method. *Journal of Biochemical Technology*.

[17] Rao CS, Prakasham RS, Lakshmi CS, Rao AB (2009). Effect of various immobilization matrices on Lactobacillus delbrucekii cells for optically pure L^+^ lactic acid production. *Current Trends in Biotechnology and Pharmacy*.

[18] Riaz A, Ul Qader SA, Anwar A, Iqbal S (2009). Immobilization of a thermostable A-amylase on calcium alginate beads from *Bacillus subtilis* KIBGE-HAR. *Australian Journal of Basic and Applied Sciences*.

[19] Dey G, Singh B, Banerjee R (2003). Immobilization of *α*-amylase produced by *Bacillus circulans* GRS 313. *Brazilian Archives of Biology and Technology*.

[20] Farag AM, Hassan MA (2004). Purification, characterization and immobilization of a keratinase from *Aspergillus oryzae*. *Enzyme and Microbial Technology*.

[21] Adinarayana K, Bapi Raju KVVSN, Ellaiah P (2004). Investigations on alkaline protease production with *B. subtilis* PE-11 immobilized in calcium alginate gel beads. *Process Biochemistry*.

[22] Sun J, Liu J, Liu Y, Li Z Optimization of entrapping conditions of nitrifying bacteria and selection of entrapping agent.

[23] Konsoula Z, Kyriakides ML (2006). Thermostable *α*-amylase production by *Bacillus subtilis* entrapped in calcium alginate gel capsules. *Enzyme and Microbial Technology*.

[24] Elibol M, Moreira AR (2003). Production of extracellular alkaline protease by immobilization of the marine bacterium *Teredinobacter turnirae*. *Process Biochemistry*.

[25] Ahmed I, Zia MA, Iqbal HMN (2011). Purification and kinetic parameters characterization of an alkaline protease produced from *Bacillus subtilis* through submerged fermentation technique. *World Applied Sciences Journal*.

[26] Morana A, Mangione A, Maurelli L (2006). Immobilization and characterization of a thermostable *β*-xylosidase to generate a reusable biocatalyst. *Enzyme and Microbial Technology*.

[27] Arya SK, Srivastava SK (2006). Kinetics of immobilized cyclodextrin gluconotransferase produced by *Bacillus macerans* ATCC 8244. *Enzyme and Microbial Technology*.

[28] Srinivasa Rao R, Borkar PS, Khobragade CN, Sagar AD (2006). Enzymatic activities of proteases immobilized on tri(4-formyl phenoxy) cyanurate. *Enzyme and Microbial Technology*.

[29] Amaral IPG, Carneiro-da-Cunha MG, Carvalho LB, Bezerra RS (2006). Fish trypsin immobilized on ferromagnetic Dacron. *Process Biochemistry*.

[30] Sayem SMA, Alam MJ, Hoq MM (2006). Effect of temperature, pH and metal ions on the activity and stability of alkaline protease from novel *Bacillus licheniformis* MZK03. *Proceedings of the Pakistan Academy of Sciences*.

[31] Çevik E, Şenel M, Abasiyanik MF (2011). Immobilization of urease on copper chelated EC-Tribeads and reversible adsorption. *African Journal of Biotechnology*.

[32] Qader SAU, Aman A, Syed N, Bano S, Azhar A (2007). Characterization of dextransucrase immobilized on calcium alginate beads from Leuconostoc mesenteroides PCSIR-4. *Italian Journal of Biochemistry*.

[33] Tanksale A, Chandra PM, Rao M, Deshpande V (2001). Immobilization of alkaline protease from Conidiobolus macrosporus for reuse and improved thermal stability. *Biotechnology Letters*.

[34] Pithawala K, Mishra N, Bahadur A (2010). Immobilization of urease in alginate, paraffin and lac. *Journal of the Serbian Chemical Society*.

[35] El-Bendary MA, Moharam ME, Ali TH (2009). Efficient immobilization of Milk clotting enzyme produced by *Bacillus sphaericus*. *Polish Journal of Food and Nutrition Sciences*.

[36] Kumar RSS, Vishwanath KS, Singh SA, Rao AGA (2006). Entrapment of *α*-amylase in alginate beads: single step protocol for purification and thermal stabilization. *Process Biochemistry*.

